# Why is NMNAT Protective against Neuronal Cell Death and Axon Degeneration, but Inhibitory of Axon Regeneration?

**DOI:** 10.3390/cells8030267

**Published:** 2019-03-21

**Authors:** Bor Luen Tang

**Affiliations:** 1 Department of Biochemistry, Yong Loo Lin School of Medicine, National University of Singapore, Singapore 117597, Singapore; bchtbl@nus.edu.sg; Tel.: +65-6516-1040; 2 NUS Graduate School for Integrative Sciences and Engineering, National University of Singapore, Singapore 117597, Singapore

**Keywords:** axon regeneration, neuroprotection, nicotinamide mononucleotide adenylyltransferase (NMNAT), nicotinamide adenine dinucleotide (NAD^+^), phosphatase and tensin homolog (PTEN), SIRT1

## Abstract

Nicotinamide mononucleotide adenylyltransferase (NMNAT), a key enzyme for NAD^+^ synthesis, is well known for its activity in neuronal survival and attenuation of Wallerian degeneration. Recent investigations in invertebrate models have, however, revealed that NMNAT activity negatively impacts upon axon regeneration. Overexpression of Nmnat in laser-severed *Drosophila* sensory neurons reduced axon regeneration, while axon regeneration was enhanced in injured mechanosensory axons in *C. elegans* *nmat-2* null mutants. These diametrically opposite effects of NMNAT orthologues on neuroprotection and axon regeneration appear counterintuitive as there are many examples of neuroprotective factors that also promote neurite outgrowth, and enhanced neuronal survival would logically facilitate regeneration. We suggest here that while NMNAT activity and NAD^+^ production activate neuroprotective mechanisms such as SIRT1-mediated deacetylation, the same mechanisms may also activate a key axonal regeneration inhibitor, namely phosphatase and tensin homolog (PTEN). SIRT1 is known to deacetylate and activate PTEN which could, in turn, suppress PI3 kinase–mTORC1-mediated induction of localized axonal protein translation, an important process that determines successful regeneration. Strategic tuning of Nmnat activity and NAD^+^ production in axotomized neurons may thus be necessary to promote initial survival without inhibiting subsequent regeneration.

## 1. Introduction

Nicotinamide mononucleotide adenylyltransferase (NMNAT) is an evolutionarily conserved rate-limiting enzyme that catalyzes the salvage pathway biosynthesis of the essential coenzyme nicotinamide adenine dinucleotide (NAD^+^) from ATP and nicotinamide mononucleotide (NMN) [1]. Humans have three NMNAT paralogues, where NMNAT1 (predominantly nuclear) and NMNAT3 (mitochondrial and cytoplasmic depending on splice variant) are more ubiquitously expressed, while NMNT2 (cytoplasmic) is enriched in brain [2,3,4]. NMNATs, as NAD^+^ synthases, have critical roles in energy metabolism. NMNAT is also a stress- and injury-induced protein [5,6] and plays a particularly critical role in the maintenance of neuronal survival and health [3,7,8]. Loss of NMNAT2 in mice resulted in perinatal lethality with severe defects in peripheral nerves and regions of the central nervous system (CNS) [9]. In fact, adequate levels of NMNAT2 appear to be critical for axon development and survival, since *NNMAT2* compound heterozygote mice present early and age-dependent peripheral nerve axonal defects [10]. Mutations in NMNAT1 in humans underlie the eye disorder Leber congenital amaurosis, which is characterized by patients suffering from early severe macular and optic atrophy [11]. In mice, overexpression of NMNAT1 protects both retinal ganglion cells and axons from glaucomatous and ischemic injury [12]. NMNAT-produced NAD^+^ has indeed been identified as a key factor that determines neuronal survival and degeneration upon insult or injury [13,14]. NMNAT and its regulation of NAD^+^ metabolism also have broader implications in terms of organismal level metabolic health and life span [15].

Axon regeneration upon injury is much more difficult in CNS neurons compared to peripheral neurons and is influenced by both external inhibitory factors within the injury environment [16], as well as by neuronal intrinsic factors and mechanisms [17,18]. Important intrinsic processes that facilitate axon regeneration include proper axonal mRNA localization, as well as adequate injury-induced local protein synthesis [19,20] and turnover [21]. In this regard, the mechanistic target of rapamycin (mTOR) [22] functions as a master regulator whose activation enhances mRNA translation and protein synthesis at the soma, as well as locally in the axon [23]. The activity of mTOR in association with the two signaling complexes in which mTOR integral part of, namely mTORC1 and mTORC2, is regulated by a complex interplay between the inhibitory tuberous sclerosis complex (TSC) family of proteins and the activating phosphoinositide 3 kinase–AKT kinase (PI3K–AKT) axis [24]. Another important player in this network is phosphatase and tensin homologue (PTEN) [25], a lipid phosphatase that antagonizes PI3K–AKT and, thus, negatively regulates mTOR signaling. PTEN is a key intrinsic inhibitor of axon regeneration, and its deletion or silencing promote regeneration of both adult peripheral neurons [26] and CNS neurons [27,28,29,30,31]. In this study, mTOR activity was conversely shown to promote axon regeneration by multiple neuron types [32,33,34,35,36]. In view of the critical and central roles played by mTOR and PTEN in axon regeneration, the action of many factors that influence the regeneration outcome would likely impinge on, or converge upon, these two molecules.

Although NMNAT is widely perceived as being critical for protection against neuronal death and degeneration, its direct influence on axon regeneration has been less clear. Recent evidence has, in fact, indicated that instead of a positive enhancement, NMNAT actually inhibits axon regeneration. In the paragraphs below, NMNAT’s known neuroprotective and anti-degenerative actions shall be briefly recapped, and recent evidence for it acting as an inhibitor of axon regeneration recounted. Plausible explanations as to why NMNAT could be neuroprotective while inhibitory of axon regeneration shall then be expounded.

## 2. NMNAT Has Important Neuroprotective Functions and Attenuates Wallerian Degeneration

There is much evidence to support the notion that NMNAT and its product NAD^+^ have key neuronal maintenance and protective functions [3,4], both during acute neuronal injury as well as in the context of neurodegenerative diseases. Other than the neuroprotective examples mentioned above, degeneration of dorsal root ganglion axons induced by rotenone, which inhibits mitochondrial electron transport and causes oxidative stresses, is delayed by NMNAT overexpression [5]. In a yeast model of proteinopathy, overexpression of the yeast homologues of NMNAT suppressed the cytotoxicity of aggregation-prone and neurodegeneration-associated polyglutamine-containing polypeptides and α-synuclein [37]. NMNAT2 is found to be downregulated prior to the onset of neurodegeneration, and its overexpression is both neuroprotective [38] and alleviates behavioral impairments [39] in mouse models of tauopathy. In this regard, NMNAT was also shown to suppress tau-induced neurodegeneration by promoting the clearance of hyperphosphorylated tau oligomers in a *Drosophila* tauopathy model [40]. In the Wobbler mice motor neuron disease model, NMNAT2 levels in the spinal cord were found to be downregulated [41]. NMNATs, particularly the brain-enriched isoform NMNAT2, thus appear to be suppressive of multiple neurotoxic agents that promote neuronal degeneration.

As far as neuronal injury is concerned, a particularly important activity of NMNAT is its attenuation of Wallerian degeneration (WD) [42]. This was first demonstrated by mice with a remarkable post-injury retardation in WD, where the underlying protective mutant gene Wallerian degeneration slow (*Wld^s^*) encodes a fusion protein of the N-terminal fragment of ubiquitination factor E4B (Ube4b) to NMNAT [43]. The NMNAT enzyme activity of the WldS protein appears to be necessary for the WD retardation phenotype [44,45]. More recent studies have delineated the role of the pro-degenerative Sterile-alpha and Armadillo motif containing protein 1 (SARM1) in WD [46]. SARM1 is required for injury-induced WD as well as axonal death by enhancing axonal NAD^+^ depletion [14,47,48], and SARM1 deficiency alleviated developmental defects and promoted survival of NMNAT2-deficient neurons [49]. Axon degeneration by injured neurons could, in fact, be blocked by NMNAT proteins that are directly transduced into transected axons [50]. NMNAT2 appears to be a labile molecule with a short half-life [7]. Maintaining a threshold level of NMNAT2 is, therefore, important for the prevention of spontaneous degeneration of healthy axons. In fact, NMNAT2 depletion alone appeared sufficient to induce WD-like features in uninjured axons, and degeneration could not be overcome by the presence of both NMNAT1 and NMNAT3 [7].

There is evidence that NMNAT’s neuroprotective function is not limited to its enzymatic activity in producing NAD^+^ [51,52]. NMNAT expression [6,52] and splicing [53] are stress-induced, and its neuroprotective action may be dependent on chaperone-like activity [4]. Some findings have indicated that while NMNAT’s enzymatic activity is necessary for neuroprotection [44,54], the resulting increase in NAD^+^ is not [54]. In fact, NMNAT1 has been shown to inhibit axon degeneration by directly blocking injury-induced and SARM1-dependent NAD^+^ destruction [55]. NMNAT could thus exerts its neuroprotective functions in different ways which could potentially compliment, or even reinforce one other.

## 3. NMNAT Inhibits Axon Regeneration in Two Invertebrate Models

Despite the overwhelming evidence for a beneficial effect for NMNAT activity in neuronal survival and attenuation of axon degeneration upon injury, two recent studies in invertebrate models have indicated that NMNAT inhibits axon regeneration. The first is reported by Chen et al. [56] based on findings in a *Drosophila* sensory neuron preconditioning injury model [57] in which axon injury ‘stabilizes’ the cell, including the entire dendritic arbor, from subsequent death and dendritic degeneration. This state of neuroprotection (NP) is accompanied by an upregulation of mitochondrial fission in dendrites and is negatively regulated by *Drosophila* caspases. NP is absolutely dependent on Nmnat, and overexpression of the latter was sufficient to induce a neuroprotective state. Interestingly, the authors found that Nmnat overexpression and reduction of the initiator caspase Dronc both inhibited axon regeneration. Likewise, Wlds has an inhibitory effect on regeneration. The basis for this Nmnat-mediated inhibition of regeneration, and whether it is dependent on NAD^+^, is unclear. However, the authors showed that the protective effect is cell autonomous through the expression of the Wlds protein in ddaC neurons, which are adjacent to ddaE neurons. The Wlds-expressing ddaC neurons formed persistent axonal stumps but did not reduce regeneration of neighboring wild-type ddaE neurons.

Using a *Caenorhabditis elegans* mechanosensory axon regeneration assay, Kim et al. [58] reported a genetic screen which identified, among others, the worm NMNAT orthologue NMAT-2 as an inhibitory protein for axon regeneration. Axon regrowth was enhanced in two independent *nmat-2*-null mutants (but not the null mutants of the paralogous *nmat-1*), as well as null mutants of another NAD^+^ synthesizing enzyme, the glutamine-dependent NAD^+^ synthase QNS-1. An enzyme active site mutant of *nmat-2* also displayed a similar phenotype, indicating that NAD^+^ is an important mediator of the inhibitory effect. The phenotype of the *nmat-2*-null mutant is rescued by a single copy of the transgene under the endogenous promoter. Interestingly, however, individual expression of the transgene in the epidermis, intestine, or mechanosensory neurons could not rescue the phenotype, which was only reversed by a combined expression in all three tissues. The authors speculated that “NAD^+^ might activate inhibitory factors in neurons and in surrounding tissues, which act together to repress the axon regenerative response” [58].

## 4. Plausible Mechanistic Explanations for the Dichotomous Effects of NMNAT

The above findings suggest NMNAT and its activity are inhibitory of axon regeneration, which appears to be somewhat counterintuitive and paradoxical. Many neurotrophic and neuronal growth factors are known to promote both neuronal survival and neurite extension. On the other hand, even if a pro-survival factor does not directly induce neurite outgrowth, it would be intuitive to assume that extended neuronal survival would likely lead to a better regenerative outcome. How could NMNAT’s neuroprotective and inhibitory axon regeneration activities be reconciled?

There are some early observations which suggest that the NMNAT activity of Wlds does not promote, and may in fact retard, axon regeneration. Regeneration of sensory axons [59] and motor axons [60] are reportedly impaired in the degeneration-delayed C57BL/Ola mouse. WD appears to be necessary for proper zebrafish trigeminal axon regeneration and innervation of the epidermis after injury [61]. The increased neurite growth that normally occurs after a conditioning lesion of dorsal root ganglion neurons was also reduced in *Wld^s^* mice [62]. The process of axonal fragmentation in WD itself, and perhaps also the subsequent clearance of the fragments by immune cells, may therefore promote neurite or axonal outgrowth. On the other hand, regeneration in terms of polarized growth of another axon could be inhibited or delayed by the persistence of the degenerating axon.

There is also the possibility that NMNAT expression in injured neurons attenuates axon regeneration in a cell non-autonomous manner. A persistent axonal stump that is slow to degrade could conceivably prevent collateral sprouts. Indeed, in *Wld^s^* mice, collateral sprouting from spared fibers after spinal cord injury was greatly reduced compared to wild-type mice, resulting in a worse locomotor recovery [63]. It is possible that NMNAT upregulates inhibitory factors, either soluble or membrane-bound, to attenuate collateral sprouting. However, this mode of action is not at work for the *Drosophila* model discussed above, where persistent stumps generated by a WldS-expressing neuron type did not reduce regeneration of different types of neighboring neurons the way NMNAT expression did.

We are, therefore, left to contemplate the possible cell autonomous mechanisms underlying the dichotomous action of NMNAT. The NAD^+^ generated by NMNAT is an essential cofactor for two major classes of proteins known to be associated with neuronal survival and degeneration. The poly (ADP-ribose) polymerases (PARPs) are a family of proteins involved in DNA repair and are most often associated with acute neuronal death and neurodegenerative diseases [64,65,66]. It was recently reported that mutations of either of two *C. elegans* PARP homologs, *parp-1* and *parp-2*, enhanced axon regeneration by injured GABA motor neurons in worms, and *PARP* silencing promoted axon regeneration of mouse cortical neurons [67]. Similar effects were demonstrated by PARP inhibitors [67]. However, another report showed that axon regeneration after optic nerve crush and spinal cord hemisection was not enhanced in *PARP* −/− mice, nor were they enhanced by treatment with the PARP inhibitor veliparib [68]. Thus, the role played by PARP in axon regeneration remains controversial.

On the other hand, we have the Sirtuin class III histone deacetylases, whose protein deacetylase activities are strictly dependent on NAD^+^ [69]. Some members of this family, particularly SIRT1, are pro-survival for neurons [70,71], and could also act as a mediator of the delay in WD mediated by NMNAT-generated NAD^+^ [72,73]. SIRT1 shuttles between the cytoplasm and the nucleus [74], and could suppress neuronal death triggered by multiple stimuli via its deacetylation of TP53 [75,76,77] and the forkhead family of transcription factors [78,79,80]. Several reports have indicated that SIRT1 promotes neurite outgrowth in cultured neural cells [81,82,83], axonogenesis of primary neurons [84] and sensory axon regeneration of adult DRG neurons [85]. However, apart from the latter example, the first two mentioned study subjects were implicated in normal healthy morphological, developmental differentiation, rather than regeneration of damaged, degenerated, or cut axons after injury. In *C. elegans*, sir-2.1 mutations did not significantly influence axon regeneration by injured GABA motor neurons, although transgenic overexpression of the Sirtuin substrate *daf-16/FOXO* did enhance axon regeneration [86].

Could SIRT1 activity actually suppress axon regeneration? As mentioned above, PTEN and mTOR are key regulators of axon regeneration [87]. Deletion of PTEN or upregulation of mTOR activity promoted regeneration even in CNS neurons [27,28,29]. Exactly how PTEN suppresses axon regeneration remains to be fully clarified, but this is likely connected to mTOR’s role in nutrient homeostasis through regulating protein translation and autophagy [88]. Axonal mRNA transport and localized axonal protein translation appear to be particularly important determinants for successful regeneration [20,21,89,90], and inhibition of these processes would retard regeneration.

**Figure 1:**
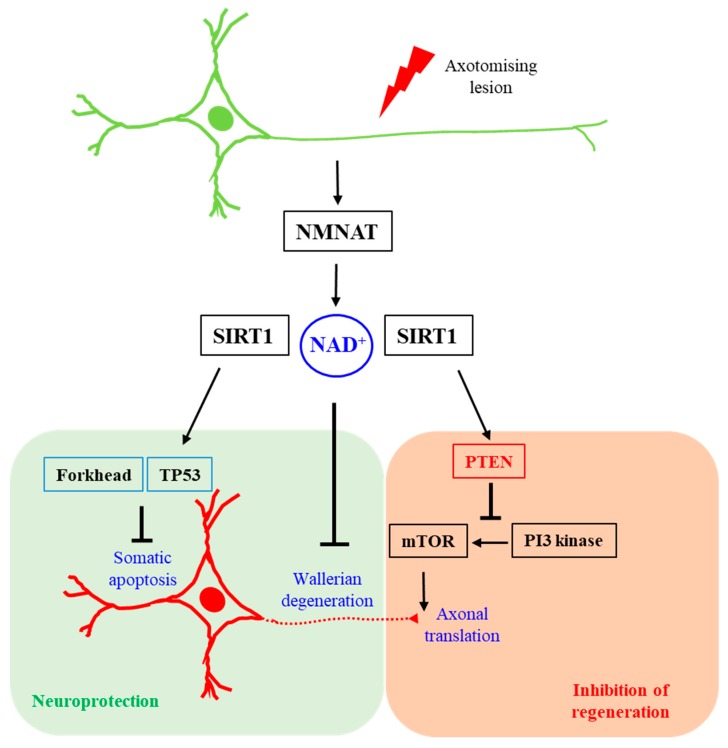
A schematic diagram illustrating how nicotinamide mononucleotide adenylyltransferase (NMNAT)-produced NAD^+^ might be protective against neuronal death and axon degeneration and simultaneously inhibitory against axon regeneration. NAD^+^ alleviates Wallerian degeneration and is a critical coenzyme of SIRT1. While SIRT1 deacetylation diminishes or modifies the death-inducing activities of TP53 and forkhead transcription factors, its deacetylation may activate the anti-axon regeneration factor PTEN, whose activity may negatively impact local axonal translation and protein turnover.

PTEN, notably, is acetylated at lysine residues (Lys125 and Lys128) within its catalytic site by P300/CREB-binding protein (CBP) associated factor (PCAF) [91], which attenuates its catalytic activity. PTEN is also acetylated at Lys402 in a manner that is dependent on CBP, and this residue lies within its carboxyl terminal PDZ domain-binding motif [92]. On the other hand, SIRT1 has been shown to be a major PTEN deacetylase [92,93], and SIRT1 deacetylation would enhance PTEN’s catalytic activity. It is, therefore, conceivable that SIRT1 activity, promoted by the NAD^+^ produced by NMNAT, could be inhibitory of axon regeneration via an enhancement of local PTEN activity. A plausible explanation of how NMNAT-generated NAD^+^ could protect against neuronal death and WD, while inhibiting axon regeneration, is summarized schematically in Figure 1 below. In brief, the activation of PTEN by NAD^+^-dependent SIRT1 deacetylation, particularly within the axon, could impinge negatively on mTOR activity, thus leading to a reduction in localized axonal protein translation, thereby attenuating axon regeneration.

## 5. Perspectives

The paragraphs above discussed recent research that supports the somewhat paradoxical observation that neuroprotective and WD-attenuating NMNAT expression or activity inhibits axon regeneration in *Drosophila* and *C. elegans* models of sensory neuron axotomy. While cell non-autonomous mechanisms could not be ruled out, a plausible cell autonomous mechanism that provides tentative explanations for the observations could be discerned. In particular, while NMNAT-produced NAD^+^ enhanced SIRT1 deacetylation of proteins that suppressed cell death and WD, its deacetylation of PTEN could, on the other hand, inhibit axon regeneration. This notion can be tested by careful assessments of possible reciprocal changes in SIRT1 and PTEN activities in NMNAT-expressing or -deleted neurons, as well as in animal models engineered to express acetylation-resistant PTEN in neurons. Of course, NAD^+^ is also important for the activities of other Sirtuin family members and, in this regard, cytoplasmic SIRT2—a major tubulin deacetylase [94] whose loss is associated with axonal degeneration [95]—would be of particular interest. A recent finding has also revealed that SIRT4 could interact with PTEN and regulate its stability in a manner that is independent of PTEN acetylation [96]. We have more to learn about how PTEN interacts with neuronal Sirtuins and how these affect its role in regeneration.

The above notion that SIRT1′s deacetylation and activation of PTEN could inhibit axon regeneration is not without caveats and reservations. There are several notable issues, including earlier findings that *C. elegans sir2.1* had little effect in axonal regeneration [86] and that SIRT1 inhibitor and its associated silencing both promoted DRG sensory axon regeneration [85]. The neuroprotective action of resveratrol, an allosteric activator of SIRT1 [97], is well known [98] and, in several cases, it has been demonstrated that it acts through SIRT1 in this regard [99,100]. Two recent reports have also indicated that resveratrol treatment promoted axon regeneration in different models [101,102]. Resveratrol has, however, a myriad of known cellular targets [103,104], and in the above cases, its role in axon regeneration was attributed to other molecules rather than SIRT1 activation. A potential rejoinder for these reservations is that SIRT1 activity is both pro-survival and inhibitory of axonal degradation, and it could indirectly contribute towards regeneration at a later time. In other words, SIRT1 activity could have both an axon regeneration-promoting effect as well as an inhibitory effect, and the outcome depends on a balance between the two.

It should also be noted that the interacting pathways involved in axon regeneration could be very much context- and neuronal type-dependent, with optimal conditions for axon regeneration differing between neuron types, for example, CNS and peripheral nervous system (PNS) neurons, or between invertebrate and mammalian neurons. Furthermore, PTEN activation and attenuation of mTOR activity could induce autophagy. The latter process is generally neuroprotective but its over-activation could also lead to neuronal cell death [105]. Autophagy has been shown to negatively regulate axon extension by primary cortical neurons [106], and even different neuron types in one region, but its induction was also found to stabilize microtubules and promote axonal regeneration in a mouse spinal cord injury model [107,108]. The above notion of PTEN mediating the axon regeneration inhibitory effect of NMNAT could, thus, be an oversimplification. In reality, axon regeneration is a complex process that is dependent on the degree of injury, the extent of neuron sparing, the types of neuron involved, and various other factors. With regards to the possibility of species differences in axonal regeneration, the process of axonal fusion that occurs spontaneously in some invertebrate species [109] would enhance nerve repair in ways that are perhaps independent of NMNAT and PTEN.

The findings discussed above also suggest that administration of restorative or regenerative strategies in neuronal injury may require more sophistication than previously perceived. One would need to strike a balance between neuronal survival/degeneration and axon regeneration, which now appear to be processes that do not go hand in hand. At the moment, it is unclear if it is possible or even beneficial to tackle the two in a temporally segregated manner, with initial neuroprotective therapeutics replaced by pro-regenerative ones after an initial acute phase of injury. Again, in this regard, we have much more to explore and learn.
